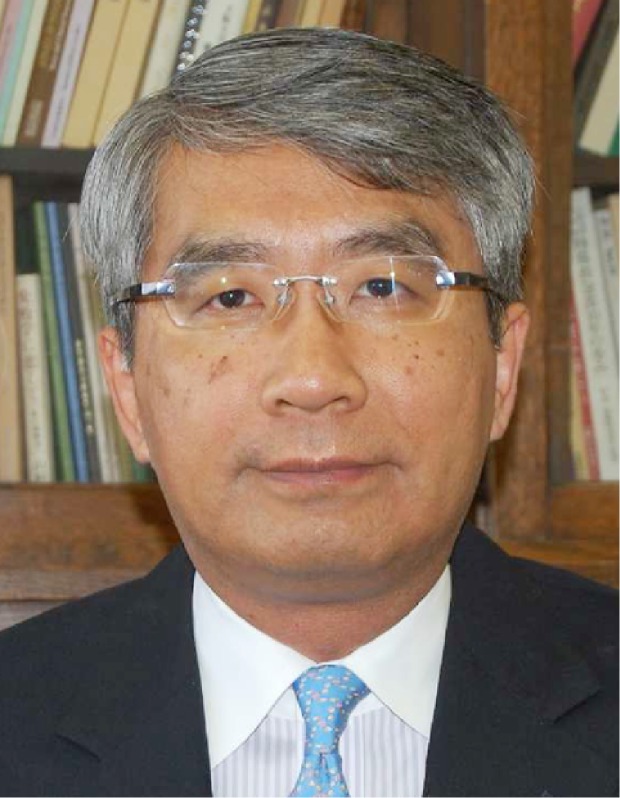# Message From the Editor-in-Chief

**DOI:** 10.2188/jea.JE20120218

**Published:** 2013-01-05

Dear Colleagues:

The official 2011 impact factor for the Journal of Epidemiology is 1.858. Our journal placed 66th among the 157 journals in the category of Public, Environmental & Occupational Health and top in the Asia-Pacific region. The proportion of self-citation was only 6%, which indicates that ours is a sound and promising journal in epidemiology. The 10 most frequently cited papers in the Journal of Epidemiology in 2010 and 2011 are shown below.

The average time between submission and first response is 27 days. For this achievement, I sincerely thank the editorial committee members and reviewers for their outstanding efforts.

In addition to our regular review articles, we published 7 joint or single review articles in 2012 for various research fields, *Epidemiology—Scientific Wisdom and Perspectives from East and West*, which are written by leading epidemiologists from Asian and Western countries. Such review series will continue this year.

Our editorial team looks forward to receiving new high-quality submissions from around the world in a broad range of topics in epidemiology. In this way, our journal can continue to advance research in basic and clinical science, public health science, and health policy.

Cordially, Hiroyasu Iso, MD, PhD, MPH Editor-in-Chief Journal of Epidemiology Professor of Public Health Osaka University Graduate School of Medicine

**Figure:**